# Identification of the risk factors for predicting severe acute kidney injury in patients after liver transplantation

**DOI:** 10.3389/fphys.2025.1614336

**Published:** 2025-07-08

**Authors:** Ran Zhou, Huan Wang, Qi Yang, Lin Han

**Affiliations:** ^1^ Department of Anesthesiology, Beijing Chao-Yang Hospital, Capital Medical University, Beijing, China; ^2^ Department of Neurology, West China School of Public Health and West China Forth Hospital, Sichuan University, Chengdu, China; ^3^ Department of Operating Room, Beijing Chao-Yang Hospital, Capital Medical University, Beijing, China

**Keywords:** liver transplantation, acute kidney injury, risk factor, restricted cubic splines, SAPS-II

## Abstract

**Background:**

Acute kidney injury (AKI) is one of the most common complications after liver transplantation (LT). Few studies have focused on the risk factors for severe AKI (KDIGO criteria: stage 3) after LT. The aim of this study was to identify critical determinants associated with the progression to severe AKI in LT patients admitted to the intensive care unit (ICU).

**Methods:**

This retrospective case–control study analyzed data from the Medical Information Mart for Intensive Care-IV (MIMIC-IV) datasets, version 3.1. Patients were categorized into two groups according to the stage of AKI. Patients diagnosed with AKI stage I or II were placed in the mild-AKI group, and the others diagnosed with AKI stage Ⅲ were placed in the severe-AKI group. Risk factors were figured out through the univariate and multivariable logistic regression models between the groups. Restricted cubic spline (RCS) analyses were conducted to determine threshold effects of the identified risk factors on severe AKI.

**Results:**

A total of 251 patients were enrolled. A total of 131 patients were diagnosed with AKI stage I or II (mild-AKI group), whereas 120 patients were diagnosed with AKI stage Ⅲ (severe-AKI group). Multivariable logistic regression analysis revealed that gender (female), total bilirubin, serum creatinine, and Simplified Acute Physiology Score II (SAPS II) were independent risk factors for severe AKI in LT patients. Male patients had a significantly lower risk of severe AKI than female patients (p = 0.023; OR = 0.349; 95% CI: 0.138–0.854). RCS analysis further revealed nonlinear associations with critical inflection points at total bilirubin 12.189 mg/dL, serum creatinine 1.118 mg/dL, and SAPS-II of 42. Beyond these thresholds, each incremental increase in these parameters demonstrated a statistically significant escalation in severe-AKI risk.

**Conclusion:**

In our study, we found that gender (female), total bilirubin (≥12.189 mg/dL), creatinine (≥1.118 mg/dL), and SAPS-II (≥42) are the independent risk factors for severe AKI in patients after LT.

## Introduction

The principal challenge in post liver transplantation (LT) care lies in achieving a critical equilibrium between immunosuppression optimization and complication mitigation while addressing systemic multi-organ interactions and chronic metabolic dysregulation. Liver transplant recipients exhibit distinct pathophysiological alterations due to immunosuppressive therapies, pharmacological burdens, and organ crosstalk, collectively predisposing them to complications such as opportunistic infections, cardiovascular/cerebrovascular events, and renal dysfunction. Acute kidney injury (AKI), a prevalent complication with reported incidence rates varying between 52% and 80%, is clinically significant due to its association with prolonged hospitalization, accelerated progression to chronic kidney disease (CKD), and diminished long-term patient survival (Hilmi et al., 2015; Milne et al., 2025).

Proactive identification of AKI risk factors enables early stratification of high-risk populations for targeted surveillance. Mechanistic elucidation of these risk determinants supports individualized therapeutic strategies to attenuate AKI incidence, optimize recovery trajectories, and enhance post-transplant quality of life. Elevated body mass index (BMI) exacerbates intraoperative hemodynamic volatility and postoperative metabolic stress, whereas preoperative hypercreatininemia reflects compromised renal functional reserve (Wu et al., 2025; Wang et al., 2025). Intraoperative hemodynamic instability, especially hypotension during the anhepatic phase, may be independently associated with AKI (Bieze et al., 2024). However, the association between intraoperative hypotension and AKI seems ambiguous. A recent cohort study revealed no significant association between hypotension exposure and postoperative AKI incidence or severity of AKI (Cywinski et al., 2024). The preservation method for *ex vivo* livers may significantly influence the incidence of post-transplant AKI through its impact on postoperative patient status. Current evidence suggests that patients in the normothermic machine perfusion (NMP) group demonstrated lower rates of both stage 1 and stage 3 AKI than those receiving conventional static cold storage (Nguyen et al., 2025). Peak lactate levels and aspartate aminotransferase (AST) elevations during time spent in the intensive care unit (ICU) demonstrate correlations with AKI severity (Fang et al., 2025; Nishino et al., 2024).

Although extensive research has investigated the risk factors for AKI following LT, the mechanisms by which these factors influence the severity of AKI progression remain poorly characterized. In this study, we aimed to find out the potential risk factors associated with severe AKI in LT patients admitted to the ICU. Furthermore, we employed restricted cubic spline (RCS) analysis to delineate nonlinear relationships and quantify threshold effects of the identified risk factors, thereby providing actionable insights for optimizing clinical management and risk stratification protocols.

## Methods

### Study design and population

This retrospective case–control study analyzed data from the Medical Information Mart for Intensive Care-IV (MIMIC-IV) datasets version 3.1 (Johnson et al., 2024; Johnson et al., 2023). MIMIC dataset is a large de-identified dataset of patients admitted to the emergency department or an ICU at the Beth Israel Deaconess Medical Center in Boston, MA. Access to the datasets was granted upon completion of the required training by one of the study authors (Ran Zhou), who received certification (certification number: 68950421).

Adult patients (age ≥18 years) who underwent LT and were transferred to the ICU after surgery between 2008 and 2022 were enrolled in this study. If a patient was transferred to the ICU more than two times, only data of the first time in the ICU were selected. Subjects who did not meet the AKI criteria (Kidney Disease: Improving Global Outcomes, KDIGO) (Durand et al., 2018) throughout the ICU period were excluded. Data entries containing >10% missing values were excluded from the final analysis. Missing information regarding the diagnosis of AKI was also excluded. Finally, 251 patients were included in this study.

Patients were categorized into two groups according to the stage of AKI. Patients diagnosed with AKI stage I or II were placed in the mild-AKI group, and the others diagnosed with AKI stage Ⅲ were placed in the severe-AKI group. The diagnosis of hypertension, type 2 diabetes mellitus (DM2), chronic kidney disease (CKD), cirrhosis, hepatitis, and stroke was based on the International Classification of Diseases codes (ICD-9/10).

### Data collection

The data included in this study mainly consist of baseline data, laboratory test results and vital signs within the first 24 h of admission to the ICU, comorbidities, treatment during the ICU stay, and prognosis. The baseline data included age, gender, weight, sequential organ failure assessment scores (SOFA), and Simplified Acute Physiology Score II (SAPS II). Laboratory results included the concentration of white blood cells (WBCs), neutrophil, lymphocytes, platelet, hemoglobin, hematocrit, albumin, sodium, potassium, total calcium, chloride, glucose total bilirubin, direct bilirubin, indirect bilirubin, alanine aminotransferase (ALT), aspartate aminotransferase (AST), blood urea nitrogen (BUN), creatinine, and the arterial blood gas results including pH, partial pressure of carbon dioxide (PCO_2_), partial pressure of oxygenation (PO_2_), lactate, total carbon dioxide (TCO_2_), anion gap, and free calcium. The results of coagulation function tests include the clotting time, fibrinogen concentration, partial thromboplastin time (PTT), and the international normalized ratio (INR). Vital signs included the heart rate (HR), the mean noninvasive blood pressure (NIBPm), respiratory rate (RR), temperature, and oxygen saturation (SpO_2_). Comorbidities included hypertension, DM2, CKE, cirrhosis, hepatitis, and stroke.

### Statistical analysis

The Shapiro–Wilk test was employed to assess the normality of continuous variables. Normally distributed variables were presented as mean ± standard deviation (SD), whereas non-normally distributed variables were expressed as median (interquartile range, IQR). Comparisons between groups for continuous variables were performed using Student’s t-test or Wilcoxon rank-sum test. Categorical variables were summarized as frequency (percentage), and group differences were analyzed using the chi-square test or Fisher’s exact test.

Variables with p < 0.05 in univariate analysis were included in multivariable logistic regression models, and odds ratios (ORs) with 95% confidence intervals (95% CI) were calculated for each variable. A p value < 0.05 was considered statistically significant.

Variables identified as potential risk factors for severe AKI in the multivariable analysis were further analyzed using RCSs to determine their threshold effects. The spline was defined using four knots at the fifth, 35th, 65th, and 95th percentiles. The threshold was determined with the OR = 1. The 95% CI was derived by bootstrap resampling.

Data analyses and figures drawing were performed with R software (version 4.4.3).

## Results

### Baseline characteristics

A total of 251 patients were enrolled in this case–control study. Among the patients, 131 patients were classified as AKI stage I or II (mild-AKI group), whereas 120 patients were diagnosed with severe AKI (severe-AKI group). The two groups did not differ significantly in age. However, the severe-AKI group had a higher proportion of female patients (28 vs. 46, p = 0.005) with comparable body weight between the groups. Height and BMI comparisons were not analyzed due to missing height data exceeding 10% of the total dataset. Notably, the severe-AKI group demonstrated significantly higher SAPS II and SOFA scores than the mild-AKI group (p < 0.001 for both). Detailed demographic and clinical parameters are summarized in Table 1.

**TABLE 1:** Basic characteristics of the included patients.

Characteristics	Overall (n = 251)	Mild-AKI group (n = 131)	Severe-AKI group (n = 120)	P
Age, years (mean (SD))	55.33 (11.76)	56.29 (11.99)	54.28 (11.46)	0.177
Gender, female, n (%)	74 (29.5)	28 (21.4)	46 (38.3)	0.005
Weight, kg (mean (SD))	87.38 (19.64)	88.07 (20.04)	86.64 (19.24)	0.566
SAPSII (mean (SD))	43.24 (11.79)	38.63 (8.67)	48.27 (12.70)	<0.001
SOFA (mean (SD))	10.59 (3.07)	9.62 (2.53)	11.65 (3.25)	<0.001
Comorbidity				
Hypertension, n (%)	71 (28.3)	44 (33.6)	27 (22.5)	0.071
DM2, n (%)	70 (27.9)	32 (24.4)	38 (31.7)	0.256
CKD, n (%)	46 (18.3)	20 (15.3)	26 (21.7)	0.252
Cirrhosis, n (%)	221 (88.0)	118 (90.1)	103 (85.8)	0.401
Hepatitis, n (%)	126 (50.2)	63 (48.1)	63 (52.5)	0.568
Stroke, n (%)	4 (1.6)	3 (2.3)	1 (0.8)	0.677
Lab analysis of the first day in ICU				
WBC, K/uL (median [IQR])	10.40 [6.75, 15.65]	10.40 [6.80, 15.40]	10.40 [6.70, 15.62]	0.931
Neutrophil, K/uL (median [IQR])	8.08 [7.24, 8.47]	8.08 [7.56, 8.08]	8.08 [7.16, 9.52]	0.101
Lymphocytes, K/uL (median [IQR])	0.70 [0.58, 0.78]	0.70 [0.65, 0.70]	0.70 [0.56, 0.98]	0.117
Platelet, K/uL (median [IQR])	79.00 [54.50, 113.50]	88.00 [59.00, 116.50]	72.00 [52.00, 100.75]	0.04
Hemoglobin, g/dL (median [IQR])	9.40 [8.30, 10.70]	9.70 [8.60, 11.30]	8.90 [8.00, 10.12]	0.001
Hematocrit, % (median [IQR])	27.90 [24.45, 31.90]	29.10 [26.00, 33.15]	26.90 [23.67, 30.15]	<0.001
Albumin, g/dL (median [IQR])	2.80 [2.50, 3.20]	2.90 [2.55, 3.20]	2.70 [2.40, 3.10]	0.021
Sodium, mEq/L (median [IQR])	138.00 [134.00, 141.00]	138.00 [136.00, 141.00]	138.00 [132.75, 141.00]	0.437
Potassium, mEq/L (median [IQR])	4.40 [4.00, 4.90]	4.30 [3.90, 4.80]	4.50 [4.10, 5.00]	0.073
Total calcium, mg/dL (median [IQR])	8.70 [8.30, 9.55]	8.70 [8.30, 9.30]	8.80 [8.30, 9.70]	0.405
Chloride, mEq/L (median [IQR])	102.00 [98.00, 105.50]	103.00 [99.00, 106.00]	101.00 [97.00, 105.00]	0.046
Glucose, mg/dL (median [IQR])	212.00 [153.00, 263.00]	217.00 [167.00, 264.00]	206.50 [140.75, 256.50]	0.093
Anion gap, mEq/L (median [IQR])	15.00 [12.00, 19.00]	15.00 [12.00, 18.00]	15.00 [12.00, 20.00]	0.252
pH (median [IQR])	7.34 [7.29, 7.39]	7.34 [7.28, 7.38]	7.34 [7.29, 7.40]	0.289
PCO_2_, mmHg (median [IQR])	42.00 [37.00, 47.00]	43.00 [37.00, 47.00]	40.00 [36.00, 46.00]	0.067
PO_2_, mmHg (median [IQR])	166.50 [95.50, 265.00]	164.00 [96.00, 278.00]	173.00 [95.75, 253.00]	0.695
Lactate, mmol/L (median [IQR])	2.75 [1.90, 4.55]	2.60 [1.80, 4.50]	3.10 [2.00, 4.53]	0.455
Total CO_2_, mEq/L (median [IQR])	23.00 [20.00, 25.00]	23.00 [20.00, 25.00]	22.00 [20.00, 24.25]	0.487
Free calcium, mmol/L (median [IQR])	1.14 [1.06, 1.22]	1.14 [1.07, 1.21]	1.14 [1.06, 1.22]	0.577
Total bilirubin, mg/dL (median [IQR])	5.50 [2.85, 10.20]	4.40 [2.40, 8.45]	6.55 [4.07, 15.25]	<0.001
Direct bilirubin, mg/dL (median [IQR])	3.75 [1.70, 6.50]	2.90 [1.50, 5.05]	4.45 [2.28, 8.22]	<0.001
Indirect bilirubin, mg/dL (median [IQR])	1.60 [0.80, 2.80]	1.50 [0.80, 2.60]	1.75 [0.88, 3.12]	0.29
ALT, IU (median [IQR])/L	401.50 [178.50, 824.00]	403.00 [202.50, 746.50]	400.75 [132.75, 930.75]	0.92
AST, IU/L (median [IQR])	639.50 [287.50, 1402.00]	645.00 [361.00, 1199.50]	634.25 [233.00, 1746.50]	0.814
BUN, mg/dL (median [IQR])	21.00 [14.00, 35.00]	18.00 [13.00, 30.50]	25.00 [15.00, 41.00]	0.002
Creatinine, mg/dL (median [IQR])	1.10 [0.90, 1.80]	1.00 [0.80, 1.30]	1.50 [1.00, 2.90]	<0.001
PT, s (median [IQR])	20.10 [17.40, 24.00]	18.80 [16.65, 22.75]	20.75 [18.35, 26.75]	0.003
Fibrinogen, mg/dL (median [IQR])	164.00 [127.50, 199.00]	170.00 [133.00, 203.50]	162.00 [118.50, 190.25]	0.073
PTT, s (median [IQR])	38.10 [34.00, 45.90]	36.70 [33.10, 42.40]	39.20 [35.08, 53.72]	0.002
INR (median [IQR])	1.80 [1.60, 2.20]	1.70 [1.50, 2.10]	1.90 [1.70, 2.42]	0.003

BUN, blood urea nitrogen; SOFA, sequential organ failure assessment scores; SAPS II, simplified acute physiology score II; WBCs, white blood cells; DM2, type 2 diabetes mellitus; CKD, chronic kidney disease; ALT, alanine aminotransferase; AST, aspartate aminotransferase; BUN, blood urea nitrogen; PCO_2_, partial pressure of carbon dioxide; PO_2_, partial pressure of oxygen; CO_2_, carbon dioxide; PTT, partial thromboplastin time; INR, international normalized ratio.

### Comorbidities and lab analysis result of the first day in the ICU

The study population demonstrated relatively high complication rates of cirrhosis (88.0%) and hepatitis (50.2%), with no significant differences observed between the two groups. Furthermore, the prevalence rates of hypertension, DM2, CKD, and stroke showed comparable distributions across both groups without statistical significance (Table 1).

The severe-AKI group exhibited significantly lower hemoglobin concentrations and hematocrit levels than the mild-AKI group. The albumin concentration was also reduced in the severe-AKI group (2.70 [2.40, 3.10] vs. 2.90 [2.55, 3.20], p = 0.021). Regarding electrolyte profiles, the severe-AKI group demonstrated higher serum potassium levels (p = 0.073) but lower chloride levels (p = 0.046). The total and direct bilirubin concentrations were significantly lower in the mild-AKI group than in the severe-AKI group. Notably, blood urea nitrogen (BUN) and creatinine levels were markedly elevated in the severe-AKI group relative to the mild-AKI group (all p < 0.05). The details of the lab analysis results are summarized in Table 1.

### Vital signs and prognosis of the patients

Among the initial vital signs recorded upon ICU admission, there were no significant differences in HR, RR, or SpO_2_ between the two groups. However, the NIBPm in the mild-AKI group was significantly higher than that in the severe-AKI group (90 [81, 100] vs. 83 [70.75, 96], p < 0.001). Additionally, body temperature in the mild-AKI group was significantly elevated compared to the severe-AKI group (37.06 [36.83, 37.33] vs. 36.92 [36.67, 37.28], p = 0.013). Regarding overall therapeutic strategies and treatment details during the ICU stay, the mild-AKI group received significantly fewer specific medications (e.g., antihypertensive agents, glucocorticoids, and immunosuppressants) than the severe-AKI group (see Table 2 for details). Due to the more severe AKI in the severe-AKI group, the duration of mechanical ventilation (49.74 [24.00, 121.60] vs. 28.45 [14.43, 52.00] hours, p < 0.001), continuous renal replacement therapy (CRRT) utilization time, the ICU length of stay, and total hospital stay were all significantly prolonged in the severe-AKI group compared to the mild-AKI group (detailed data in Table 2). Five patients died in the ICU in the severe-AKI group.

**TABLE 2:** Vital signs and prognosis of the patients.

Vital signs/Prognosis	Overall (n = 251)	Mild-AKI group (n = 131)	Severe-AKI group (n = 120)	P
HR, bpm (median [IQR])	92.18 (17.49)	92.60 (17.17)	91.71 (17.90)	0.686
NIBPm, mmHg (median [IQR])	87.00 [76.50, 98.00]	90.00 [81.00, 100.00]	83.00 [70.75, 96.00]	<0.001
RR, insp/min (median [IQR])	18.00 [15.00, 20.00]	18.00 [15.00, 20.50]	18.00 [15.00, 20.00]	0.748
SpO_2_, % (median [IQR])	100.00 [98.00, 100.00]	100.00 [98.00, 100.00]	100.00 [98.00, 100.00]	0.466
Temperature, °C (median [IQR])	37.00 [36.72, 37.33]	37.06 [36.83, 37.33]	36.92 [36.67, 37.28]	0.013
Anti-HT, mg (median [IQR])	247.80 [120.00, 472.50]	247.80 [95.00, 275.00]	255.00 [207.50, 602.50]	<0.001
Glucocorticoids, mg (median [IQR])	122.50 [91.75, 174.35]	110.00 [90.00, 145.00]	137.50 [94.38, 190.00]	0.002
Immunosuppressant, mg (median [IQR])	4,032.00 [3025.50, 5811.75]	3539.00 [3023.25, 5068.25]	4,557.25 [3027.00, 6100.88]	0.048
Ventilation, n (%)	226 (90.0)	115 (87.8)	111 (92.5)	0.301
Ventilation time, h (median [IQR])	40.87 [18.32, 81.94]	28.45 [14.43, 52.00]	49.74 [24.00, 121.60]	<0.001
CRRT, n (%)	71 (28.3)	1 (0.8)	70 (58.3)	<0.001
CRRT, d (median [IQR])	0.00 [0.00, 2.50]	0.00 [0.00, 0.00]	3.00 [0.00, 7.00]	<0.001
Sepsis, n (%)	217 (86.5)	110 (84.0)	107 (89.2)	0.309
Hospital stay, d (median [IQR])	24.13 [11.23, 44.10]	16.66 [9.38, 31.84]	36.98 [17.41, 55.10]	<0.001
ICU stay, d (median [IQR])	3.64 [2.24, 6.32]	2.78 [1.94, 3.87]	5.50 [3.11, 10.47]	<0.001
Death in the ICU, n (%)	5 (2.0)	0 (0.0)	5 (4.2)	0.056

HR, heart rate; NIBPm, the mean noninvasive blood pressure; RR, respiratory rate; SpO_2_, temperature and oxygen saturation; HT, hypertension; CRRT, continuous renal replacement therapy; ICU, intensive care unit.

### Multivariable logistic regression and RCS results

**FIGURE 1:**
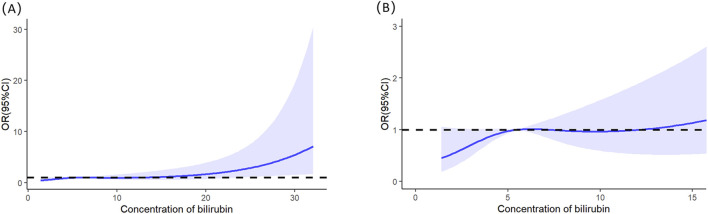
Restricted cubic splines of the total bilirubin on severe AKI. **(A)** Overall results of the whole patients. **(B)** Result of the local magnification after adjusting the horizontal and vertical coordinates.

**FIGURE 2:**
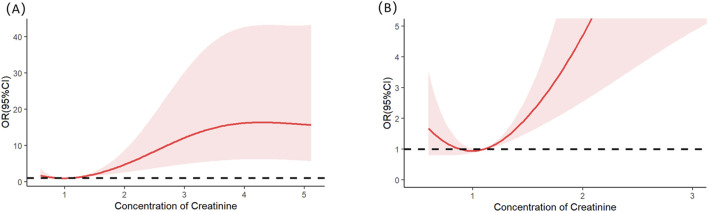
Restricted cubic splines of the serum creatinine on severe AKI. **(A)** Overall results of the whole patients. **(B)** Result of the local magnification after adjusting the horizontal and vertical coordinates.

**FIGURE 3:**
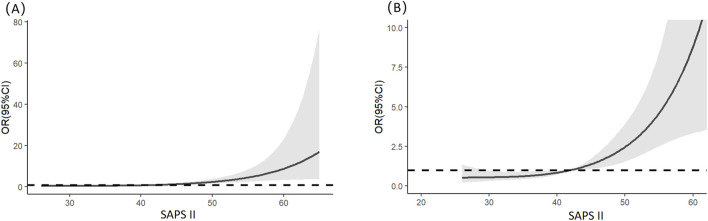
Restricted cubic splines of the SAPS II on severe AKI. **(A)** Overall results of the whole patients. **(B)** Result of the local magnification after adjusting the horizontal and vertical coordinates.

Multivariable logistic regression analysis revealed that gender, totalbilirubin, serumcreatinine, and SAPSII were independent risk factors for severe AKI in LT recipients (see details in Table 3). Specifically, male patients had a significantly lower risk of severe AKI than female patients (p = 0.023; OR = 0.349; 95% CI: 0.138–0.854). For continuous variables (total bilirubin, serum creatinine, and SAPS II), RCS analysis was utilized to further clarify their threshold effects on severe-AKI risk. The RCS results demonstrated the following trends: for total bilirubin, the OR was 1.002 at a concentration of 5.56 mg/dL, 1.003 at 7.103 mg/dL, and 1.002 at 12.189 mg/dL, with a significant increase in severe-AKI risk observed when total bilirubin exceeded 12.189 mg/dL (Figure 1). Similarly, for serum creatinine, the OR was 1.020 at 0.868 mg/dL and 1.020 at 1.118 mg/dL, indicating a marked elevation in severe-AKI risk when creatinine levels surpassed 1.118 mg/dL (Figure 2). For the SAPS II score, the OR was 1.007 at a threshold value of 42 (Figure 3).

**TABLE 3:** Risk factor analysis after multivariable logistic regression.

Risk factor	B	SE	z	P	OR	95% CI
Gender	−1.052	0.462	−2.276	0.023	0.349	(0.138, 0.854)
Total bilirubin	−0.065	0.032	−2.047	0.041	0.937	(0.877, 0.995)
Creatinine	−0.543	0.208	−2.609	0.009	0.581	(0.368, 0.846)
SAPS-II	−0.081	0.021	−3.840	<0.001	0.923	(0.884, 0.960)

Adjusted by height, platelet, hemoglobin, albumin, chloride, indirect bilirubin, blood urea nitrogen, PT, PTT, INR, NIBPm, temperature, antihypertensive medicine, glucocorticoids, immunosuppressant, and SOFA.

## Discussion

In the postoperative management of liver transplant patients within the ICU, prevention of non-hepatic complications remains a critical focus, with AKI emerging as a frequent and prognostically significant complication. Current clinical practice indicates that only severe AKI (stage ≥3) typically requires CRRT, and these patients exhibit markedly worse outcomes than those with mild-AKI (stages 1–2). To investigate risk stratification for severe AKI in this population, we conducted a real-world data analysis using the MIMIC-IV database. After screening 251 confirmed cases of AKI after LT, a case–control study design was implemented to compare clinical characteristics between mild-AKI (stages 1–2) and severe-AKI (stage ≥3) groups. Multivariate logistic regression identified gender (female), total bilirubin, creatinine, and SAPS-II score as independent risk factors for severe AKI. RCS analysis further revealed nonlinear associations with critical inflection points at total bilirubin 12.189 mg/dL, serum creatinine 1.118 mg/dL, and SAPS-II score of 42. Beyond these thresholds, each incremental increase in these parameters demonstrated a statistically significant escalation in severe-AKI risk, providing clinically relevant benchmarks for early identification of high-risk patients requiring intensified renal surveillance and preventive measures in ICU management.

This study identified female gender as an independent risk factor for severe AKI following LT. One of the causes of AKI after liver transplantation is renal ischemia–reperfusion injury. Gender differences in AKI may be related to endogenous estrogen suppressing the renal sympathetic nervous system and reducing the local norepinephrine level (Ma et al., 2021). In addition, women are estimated to have fewer glomeruli than men. A reduction in the nephron number has a significantly reduced renal functional reserve and is more susceptible to kidney injuries of hypotension or contrast volume (Luyckx et al., 2011). Similarly, a 2015 investigation reported a relatively higher AKI risk in female LT recipients, however with distinct methodological focus (Hilmi et al., 2015). Whereas the prior study analyzed risk factors for AKI development, our research specifically examined severity stratification (mild vs. severe AKI) among established AKI cases. Furthermore, in 4,676 patients with myocardial infarction who underwent percutaneous coronary intervention, AKI occurred more often in women than in men (Kanic et al., 2018). Yichuan Wang et al. (2023) found that women display increased AKI severity after cardiac surgery and they thought that the lower BMI in females may contribute to a different risk profile for AKI compared to males because the lower BMI may affect the distribution and metabolism of medications, including nephrotoxic agents, which can impact the occurrence of AKI. These studies demonstrate complementary and progressive scientific exploration, with congruent data trends collectively substantiating the consistent clinical observation of heightened AKI susceptibility in female patients.

The development of AKI following LT arises from a multifactorial etiology involving pre-, intra-, and postoperative variables, as well as donor–recipient factors. For example, hypotension during the anhepatic phase may result in AKI (Bieze et al., 2024). LT recipients with the presence of chronic HCV infection showed a more frequent development of AKI (Vijayakumar et al., 2024). At the end of surgery, cystatin C levels showed a better predictive value and higher accuracy in identifying AKI (Kim et al., 2024). Meanwhile, a high D-dimer-to-fibrinogen ratio was independently associated with AKI (Park et al., 2024). This study specifically focused on screening clinical parameters within 24 h of ICU admission postoperatively. Postoperative hepatic dysfunction inevitably elevates the risk of complications, with our study identifying elevated total bilirubin levels as an independent risk factor for severe AKI, demonstrating a linear dose–response relationship between bilirubin elevation and AKI risk. These findings align with previous research reporting postoperative AST elevation as another established risk factor for AKI development in this clinical context (Huang et al., 2025).

Serum creatinine is a core biomarker for assessing glomerular filtration function. Previous studies have established that elevated serum creatinine concentrations can predict AKI across various disease types (Wu et al., 2025; Jiang S. et al., 2025; Jiang W. et al., 2025). Few predictive model developments have focused on determining the creatinine threshold associated with a significant elevation in severe-AKI risk, as explored in this study. Our findings identified that a creatinine concentration exceeding 1.118 mg/dL correlates with increased severe-AKI risk, a critical threshold that merits further comparative validation in subsequent research.

The SAPS-II developed by Jean-Roger Le Gall et al. in 1993 is a simplified acute physiological scoring system designed to assess disease severity and predict in-hospital mortality in ICU patients, enhancing clinical practicality through streamlined parameters such as physiological indicators, age, and comorbidities (Le Gall et al., 1993). Our study found that when the SAPS-II score exceeds 42 points, the risk of severe AKI progressively increases with higher scores. Additionally, research by Tian et al. (2024) demonstrated that the SAPS-II score serves as an independent risk factor for AKI development in patients with liver cirrhosis. These findings collectively confirm the validity of the SAPS-II score in predicting AKI, which warrants heightened attention from ICU physicians to optimize early risk stratification and clinical interventions.

Urinary biomarkers may offer superior predictive value for early identification of severe AKI (Lima et al., 2024). However, this study was constrained by insufficient acquisition of valid urinary composition data, thereby precluding in-depth analysis of this parameter. This constitutes a methodological limitation of the current investigation, which will be systematically addressed in subsequent research through rigorous protocol optimization and enhanced biological sample collection. Furthermore, this study is a retrospective case–control study, and as the data were not collected prospectively, some data are missing, which may partially impact the results. Additionally, although this real-world study features authentic data, the sample size is relatively small, and the findings lack external validation. Further investigations are warranted in subsequent research.

## Conclusion

In our study, we found that gender (female), total bilirubin (≥12.189 mg/dL), creatinine (≥1.118 mg/dL), and SAPS-II (≥42) are the independent risk factors for severe AKI in patients after LT.

## Data Availability

The original contributions presented in the study are included in the article/supplementary material; further inquiries can be directed to the corresponding author.
